# A prospective natural history study of post acute sequalae of COVID-19 using digital wearables: Study protocol

**DOI:** 10.21203/rs.3.rs-3694818/v1

**Published:** 2023-12-07

**Authors:** Sherine El-Toukhy, Phillip Hegeman, Gabrielle Zuckerman, Roy Das Anirban, Nia Moses, James F. Troendle, Tiffany M. Powell-Wiley

**Affiliations:** National Institute on Minority Health and Health Disparities; National Institute on Minority Health and Health Disparities; National Institute on Minority Health and Health Disparities; Biostrap USA, LLC; National Institute on Minority Health and Health Disparities; National Heart Lung and Blood Institute; National Heart Lung and Blood Institute

**Keywords:** post-acute sequelae of COVID-19, wearable devices, physiological parameters, natural history, prospective cohort

## Abstract

**Background:**

Post-acute sequelae of COVID-19 (PASC) is characterized by having 1 + persistent, recurrent, or emergent symptoms post the infection’s acute phase. The duration and symptom manifestation of PASC remain understudied in nonhospitalized patients. Literature on PASC is primarily based on data from hospitalized patients where clinical indicators such as respiratory rate, heart rate, and oxygen saturation have been predictive of disease trajectories. Digital wearables allow for a continuous collection of such physiological parameters. This protocol outlines the design, aim, and procedures of a natural history study of PASC using digital wearables.

**Methods:**

This is a single-arm, prospective, natural history study of a cohort of 550 patients, ages 18 to 65 years old, males or females who own a smartphone and/or a tablet that meets pre-determined Bluetooth version and operating system requirements, speak English, and provide documentation of a positive COVID-19 test issued by a healthcare professional or organization within 5 days before enrollment. The study aims to identify wearables collected physiological parameters that are associated with PASC in patients with a positive diagnosis. The primary endpoint is long COVID-19, defined as ≥ 1 symptom at 3 weeks beyond first symptom onset or positive diagnosis, whichever comes first. The secondary endpoint is chronic COVID-19, defined as ≥ 1 symptom at 12 weeks beyond first symptom onset or positive diagnosis. We hypothesize that physiological parameters collected via wearables are associated with self-reported PASC. Participants must be willing and able to consent to participate in the study and adhere to study procedures for six months.

**Discussion:**

This is a fully decentralized study investigating PASC using wearable devices to collect physiological parameters and patient-reported outcomes. Given evidence on key demographics and risk profiles associated with PASC, the study will shed light on the duration and symptom manifestation of PASC in nonhospitalized patient subgroups and is an exemplar of use of wearables as population-level monitoring health tools for communicable diseases.

**Trial registration:**

ClinicalTrials.gov NCT04927442

## Introduction

Long-term health consequences have been identified as a core outcome domain for studies of COVID-19 patients.^1^ Dubbed as “long covid” and its sufferers as long haulers,^2^ post-acute sequelae of COVID-19 (PASC) has been described on social media and in news media.^3,4^ These reports referred to experiences of patients who take longer-than-normal time to recover or have 1 + symptoms that persist or develop post recovery.^5,6^ This prompted calls to investigate “prevalence, type, duration, and severity of persistent symptoms following resolution of acute SARS-CoV-2 infection, [and] risk factors associated with their development.”^7^ Definitions of PASC vary depending on onset and duration of symptoms, number and clustering of symptoms, patient population, and possibility of alternative explanations.^8–11^ Heterogeneity in defining PASC translates into varied prevalence estimates, ranging from 43–80% in systematic reviews and meta-analyses of PASC among COVID-19 patients at ~ 1 + month post infection.^12–15^ Currently, WHO defines PASC as a “condition … in individuals with a history of probable or confirmed SARS CoV-2 infection, usually 3 months from the onset of COVID-19 with symptoms and that last for at least 2 months and cannot be explained by an alternative diagnosis.”^16^

Patient-reported outcomes (PROs) and clinical evaluations show COVID-19 patients exhibit long-term health complications, regardless of initial disease severity,^17–21^ attributed to several hypothesized biological mechanisms (e.g., autoimmune and inflammatory responses).^11,22,23^ PASC symptoms include fatigue, memory loss, and chest and abdominal pain, among 200 + others that affect multiple organs/systems,^12,24–26^ whereas emergent conditions include diabetes and hypertension.^27,28^ Corroborating evidence shows persistent symptoms in recovered patients of other coronaviruses^29,30^ and post-viral chronic syndromes (e.g., ME/CFS).^31^ Evidence of PASC is based on data primarily from hospitalized^18,19,32–35^ and minimally from nonhospitalized patients,^17,36^ who represent ~ 20% and 80% of COVID-19 patients, respectively.^37^ Longitudinal studies of PASC are limited because they have single-time outcome measurements,^15^ or sparsely spaced data collection intervals, and/or short follow-ups (e.g.,^32,34,38,39^). Furthermore, studies focus on single symptoms or symptom clusters (e.g.,^40^), while few report psychological and well-being outcomes (e.g.,^18,33–35,41^). This raises the need for prospective longitudinal studies of nonhospitalized COVID-19 patients with extended follow-ups to understand the nature, duration, and multi-symptom manifestation of PASC.^42,43^

Wearable devices continuously capture physiological parameters, which have been beneficial in detecting COVID-19 and monitoring its acute-phase symptoms.^44–59^ These studies show that physiological, sleep, and activity parameters differ in the pre-infection vs. acute phases of the disease, symptomatic vs. asymptomatic patients, and COVID-19 patients vs. healthy people. Physiological parameters collected via wearables parallel clinical indicators (e.g., elevated respiratory rate)^60^ and self-reported symptoms (e.g., shortness of breath)^25^ documented in patients and are associated with PASC.^61,62^ Wearables-based studies of PASC are scarce and have short follow-ups.^63,64^ COVID-19 wearables studies have several limitations including focus on select parameters (e.g.,^54,64^); use of custom or specialized devices (e.g.,^59,65^), which limits their applicability and population level impact; or recruitment of participants who already own wearable devices (e.g.,^50,54^), thereby introducing between-device variations in parameter calculations and self-selection biases.^66^

This protocol outlines the design, objectives, and procedures of a 6-month decentralized natural history study of PASC in a cohort of 550 nonhospitalized patients with a confirmed positive COVID-19 diagnosis.

## Methods

### Design.

This is a single-arm, prospective, observational study of PASC in a cohort of 550 nonhospitalized patients with a positive COVID-19 diagnosis.

### Objective.

The study aims to identify physiological parameters collected by wearable devices that are associated with PASC.

### Endpoints.

The primary endpoint is long covid, defined as ≥ 1 symptom at ≥ 3 weeks from date of first symptom onset or positive diagnosis, whichever comes first. The secondary endpoint is chronic covid, defined as ≥ 1 symptom at ≥ 12 weeks from symptom onset or positive diagnosis.^6,9–11^

### Hypothesis

There is an association between physiological parameters collected by wearable devices and patient-reported long/chronic covid.

### Population.

A total of 550 patients will be recruited based on the demographic composition of the US population (Table 1).^67^ A prospective research volunteer (PRV) must meet all the following inclusion criteria to be eligible for enrollment:

1. Adult males or females, ages 18 to 65. The pediatric population < 18 years is excluded because it differs from adults 18 + years in infection rates, symptom manifestation, and outcomes.^68^ The > 65 years population is excluded because of its high-risk status, which prompted protections to reduce transmission among the elderly, especially in nursing homes.^69,70^
2. Documentation of a SARS-Cov-19 PCR or antigen/rapid positive test, ≤ 5 days before enrollment, issued by a public or private healthcare or COVID-19 testing facility, provider, or practitioner.
3. Ownership of or access to a smartphone/tablet, with an existing cellular data plan, that is compliant with the following specifications: (a) Android devices with an operating system of 6.0 or newer; Bluetooth 4.2, 5.×, or 6.0 and that implement BLE standard, (b) iPhone 6s/6s plus or newer, (c) iPad mini 4 or newer, iPad 5th generation or newer, iPad Air 2 (2nd generation or newer), iPad Pro (9.7”, 10.5”, 11” 1st generation or newer), 12.9-inch iPad Pro 1st generation or newer.
4. English language proficiency. Non-English speakers will be unable to use the study mobile application, which is available only in English.
5. Ability to understand and willingness to consent to participate.
6. Stated willingness to comply with all study procedures and availability for study duration.
7. Agreement to adhere to lifestyle considerations throughout the study (e.g., wear wristband and temperature patch).

An individual who meets any of the following exclusion criteria is not eligible to enroll: (1) residence outside of US mainland, (2) enrollment in clinical trials on experimental COVID-19 therapeutics at baseline, (3) requirement of hospitalization at enrollment, (4) known history of allergic reaction to adhesives, and (5) inability to consent and unwillingness to comply with study procedures (e.g., downloading a mobile application and sharing the data with research team, providing required data such as close kin contact information).

### Recruitment.

The Office of Patient Recruitment (OPR) created all recruitment materials and advertised the study online,^71^ in print, and on NIH clinical center television monitors. Commercial marketing venues are used depending on recruitment pace and funding availability. Additionally, we partnered with Fulgent Genetics, a company that administers COVID-19 testing in LA, California. As part of their online scheduling for COVID-19 testing, patients who express interest in participating in research studies are asked “would you be interested in sharing information you submitted during this registration process with researchers so they can contact you about studies?” We access information about patients who have agreed to be contacted. We engage in outreach activities to increase enrollment of minorities.

### Study procedures.

PRVs contact OPR’s recruitment center by phone or email. OPR’s staff screens interested PRVs for initial eligibility using yes/no questions. OPR staff shares basic demographic and contact information of eligible PRVs with the study team. PRVs who reach out directly to us or are part of Fulgent Genetics’ patient database receive an email and text invitation to enroll in the study, which include a link to a proactive recruitment survey with yes/no questions like those that OPR uses to determine initial PRV eligibility. These initial screening questions are about (a) PRVs age, (b) having a confirmed positive COVID-19 test within the past 72hrs, which leaves us two additional days to enroll PRVs within the 5-day eligibility window, (c) having an Apple or Android smartphone or tablet with brand and model information, and (d) willingness to comply with study procedures. Screening activities are pre-consent.

PRVs who pass initial screening receive a welcome email inviting them to schedule an enrollment appointment. The email includes a link to an electronic informed consent (IC), which states this is a research study and outlines the schedule of activities (Table 2), risks and benefits to patients, and the voluntary and confidential nature of participation. The IC emphasizes that the study wearable devices should not be the basis of any medical decisions. During the appointment, we review the IC with patients who are given opportunities to ask questions and request time to consult with their families or physicians before consenting to participate. Patients who e-consent to participate undergo eligibility verification and are enrolled only if they meet all inclusion criteria and none of the exclusion criteria. Patients who have ≤ 4GBs/person on their monthly cellular data plan and limited access to personal wifi are provided $60 gift cards to purchase additional GBs from their phone carrier.

Upon enrollment, patients are assigned a study ID and mobile application login credentials, which ensures patients do not use PII to create an application account. Using the enrollment date, REDCap generates calendar dates that correspond to pre-loaded randomized data collection schedules that map the days on which each patient must perform study activities over six months (Table 3). The first day of device wearing starts on the third calendar day post enrollment to allow time for shipping an enrollment kit. During the first month, patients are required to wear a wristband and a temperature patch around-the-clock and answer a daily two-question ecological momentary assessment (EMA). For months 2 to 6, patients are required to wear a wristband only for randomly selected 20 pairs of two consecutive days, resulting in additional 40 total device-wearing days. The schedule has a minimum of 48hrs buffer between two consecutive device-wearing periods, an approximately equal representation of weekdays and weekends where each day of the week appears 5- or 6-times during months 2 to 6, and an equal number of device-wearing days each month (i.e., 8 days). For each 48-hour device-wearing period, patients answer a two-question EMA. Patients are encouraged to wear the wristband and temperature patch upon receiving the enrollment kit, to wear the wristband beyond the 8 scheduled days per month during months 2 to 6, and to make up missed device-wearing days.

Patients are asked to complete one baseline and six monthly surveys. The baseline does not expire but patients are encouraged to complete it instantly after their enrollment appointment. Monthly surveys remain open for 12 days. Patients receive their data collection schedule, mobile app login credentials, and link to baseline survey during their enrollment appointment. This allows us to verify patients are receiving our electronic communications and to review these elements with them. We overnight ship each patient an enrollment kit, which includes (a) print copy of data collection schedule and their username for the mobile application account, (b) wristband, charger, and sweatband, (c) temperature patch, charger with batteries, and adhesives to secure the patch onto one’s body, (d) information sheet with first-time device set up instructions, data sync and troubleshooting tips, (e) clinical trials informational sheet, and (f) a pre- paid return slip with complete address information to ship back any defective devices.

For patients with no incoming wearables data and EMA responses for three consecutive days during the first month or two consecutive data collection periods during months 2 to 6, we attempt to contact patients to counsel them on the importance of adhering to their assigned study schedule. If we fail to reach a patient, we contact their close kin to complete an adverse events survey. Hospitalized patients are allowed to resume their participation in the study upon discharge and will complete an adverse events survey on their hospital stay. Upon completing the study, patients receive an end-of-study email thanking them and instructing them on how to create a personal mobile application account to continue using their wearable device should they choose to. After data analysis is complete, patients will receive a summary of main findings.

### Study evaluations.

For six months, enrolled patients engage in various research activities.

1. Physiological parameters are passively collected through a wristband, Biostrap EVO Biometric set^72^ and a VIVALINK FDA-cleared temperature monitor.^73^ The wristband is a non-invasive red and infrared optical sensor, which is less sensitive to skin tone variations and perfusion levels, and includes built-in accelerometer and gyroscope sensors that capture 6-axis motion data. Raw biosignals are used to estimate the following: (a) activity tracking accelerometer data captured continuously at 10HZ and converted into step count and activity duration saved in one-minute increments, (b) arterial pulse volume monitoring using PPG, a 45-second recording collected at 43HZ, which provides derived biometrics including HR, HRV with RR times output, SpO_2_ as percent oxygen, and respiratory rate, and (c) sleep analysis (e.g., sleep duration, sleep onset and wake time, sleep stages). The wristband technology has been validated previously.^74^ The temperature patch collects body temperature captured continuously and reported as an average value each minute given the smartphone is within range. To ensure consistency in patient data, we set the sampling frequency to every 5 minutes and hid the settings option that allow patients to adjust the rate, duration, and intervals between measurements on the mobile application. Patients have instant and continuous access to their data.
2. EMAs capture COVID-19 symptoms and medications^75,76^ that are collected in the mobile application (Table 4).
3. Baseline survey that captures demographics and social determinants of health,^75–77^ technology access,^78,79^ COVID-19 symptoms and medications,^75,76^ medical information,^75,76^ risk and protective factors,^78^ and PROs that capture cognitive, mental, and physical health domains.^80–89^
4. Monthly surveys repeatedly capture data collected at baseline, mainly COVID-19 symptoms and medications;^75,76^ medical information;^75,76^ and PROs.^80–89^ The first and last monthly surveys capture wearables acceptability data.^90–92^ The last survey captures updated data on COVID-19 re-infection, vaccination, and medical care since enrollment in the study.
5. Adverse outcomes survey captures the occurrence and timing of death or hospitalization, length of hospital stay, and treatment received.^75,76^

Baseline, monthly, and adverse outcomes surveys are collected using REDCap electronic data capture tools hosted at NIDDK.^93,94^ REDCap is a secure, web-based platform that serves as the project database and data capture tool. REDCap houses the IC and other study forms (e.g., eligibility verification). The BTRIS REDCap team of the NIH Clinical Center provided REDCap project administration and consultation.

### Compliance and retention.

To maximize compliance with study activities, automatic reminders are sent to all patients. Additional reminders are sent to non-compliant patients. To maximize retention, we review an infographic aimed at increasing patients’ research literacy and highlighting the importance of completing research studies during their enrollment appointment.^95^ Additionally, whenever there are 50 actively enrolled patients in a given month, protocol compliant patients who completed their most recent monthly survey and scored ≥ 75% for device wearing and EMA completion are entered in monthly raffles for a $100 gift card. Patients keep the wearable devices as a participation incentive.

### Statistical Analysis.

We assume 20%–35% event rates for the primary outcome of long covid and 10% event rate for the secondary outcome of chronic covid.^17,96^

#### Power.

A non-probability sample of 550 COVID-19 patients will be recruited for this study. With 550 participants, we have >85% power to detect a standardized difference (δ/σ) of 0.28, 0.33, and 0.43 in daily continuous physiologic parameters between long-or-chronic-covid and non-long-covid groups, assuming 35%, 20%, or 10% of participants will have long-or-chronic-covid, respectively, and allowing a two-sided type I error of 5%. Our analysis will use multiple days of data per participant and will adjust for prognostic baseline factors, thus power will be substantially higher.

### Analysis plan.

We will use a linear mixed model of daily awake-time and sleep-time (calendar day) values of physiological parameters to determine associations with long and chronic covid, the primary and secondary endpoints. All valid days will be included for all subjects where a valid day consists of 18hrs of device wearing. Models will include baseline fixed-effect factors for demographic and clinical variables. A term for COVID-19 status group (i.e., non-long, long-covid, chronic-covid) will be included. All models will have a fixed-effect cubic model for time since testing positive or symptom onset for each COVID-19 status group. Subject-specific random effects (intercept, slope) and a spatial (in time) model will be used for within-subject errors, accounting for correlation between observations within a subject. The cluster robust (CR2) variance will be used. Results of the above model will allow plotting differences between COVID-19 status groups at any time. For formal testing between groups, we will fit models exactly as described above, but with only linear time models for each COVID-19 status group. Contrasts between the slope terms will then be used to test if groups have different trends over 6 months. Linear mixed-effect models are robust to missing data based on a missing-at-random assumption.^97^ Sub-group analyses will be performed for analyses of both primary and secondary events restricted to patients within demographic characteristics (e.g., race/ethnicity).

### Data security.

Data from wearables are transferred to iOS or Android mobile phones via Bluetooth and stored in Biostrap cloud servers for data processing to estimate physiological parameters. Patients’ PII and PHI are not shared with Biostrap. Data are downloaded weekly to study server at NIMHD and a final data transfer will occur at study end.

## Discussion

This protocol is of a prospective, natural history study of PASC among a cohort of 550 non-hospitalized with confirmed positive COVID-19 diagnosis. The study captures intensive longitudinal physiological data collected via digital wearables and PROs for six months. The study will shed light on the course of PASC in nonhospitalized patients and represents an example for use of wearable technology in disease monitoring generally and COVID-19 specifically.

Despite the pandemic emergency state declared officially over,^98^ research into PASC remains a priority given its health and economic costs,^99,100^ national initiatives,^101,102^ continued scientific interest,^103,104^ and patient advocacy.^105–107^ Conservatively, there are 10 to 30 million people who experience PASC, with estimates likely higher if probable and suspected cases are included beyond the 100 + million confirmed COVID-19 cases in the US.^108^ The study will shed light on PASC in patient subgroups given its associations with demographics and risk profiles.^34^ This is particularly important because evidence on PASC in racial/ethnic groups is scarce and inconclusive^11,17,40,61,109^ despite minorities disproportionally bearing the burden of COVID-19 infection and adverse outcomes.^110^ While COVID-19 wearables-based studies focused mainly on its detection or acute phase, we capture a multitude of PASC digital biomarkers. This will allow us to identify PASC digital phenotypes that aid in its clinical assessment and treatment.^111,112^ Furthermore, we can triangulate evidence of PASC from physiological parameters and self-reported patient outcomes.^113^

The study’s wider public health implications pertain to the role of mobile technologies in digital medicine.^111,112,114,115^ Indeed, mobile technologies have been central to public health response to the COVID-19 pandemic^116,117^ –from crowdsourcing platforms for contact tracing^118^ to symptom reporting^119–121^ and sensor data collection.^104,122^ Wearables physiological data improve the identification of COVID-19 positive cases above and beyond self-reported symptoms,^50^ which have been the basis of pilot COVID-19 detection tools,^46–48^ and show preliminary differentiation between COVID-19 and other respiratory illnesses.^123^ Prior to COVID-19, wearables have been piloted for the detection, monitoring, and public surveillance of communicable and noncommunicable diseases, health conditions, and behaviors.^124–131^ They enjoy increasing uptake and acceptability, and afford opportunities for unobtrusive and continuous capture of objective data, include awake- and sleep-time data, outside of traditional healthcare settings.^111,132^ When integrated with remote monitoring platforms, patient-generated data can support real-time feedback to patients and physicians that is calibrated to a patient’s baseline data and has the potential to improve individual-level health outcomes^133,134^ (cf^135^). If widely adopted, wearables can be scaled up as cost-effective tools for population-level disease surveillance, management, and prevention.^136^ Noteworthy, evidence on wearables and their integration in clinical practice is in its infancy and challenges exist until their potential is fully realized.^134,136,137^

Study strengths include its intensive longitudinal data on PROs and physiological parameters, six-month follow up, and focus on nonhospitalized patients whose characteristics and disease trajectory differs from hospitalized patients^13,138,139^ (cf^12^). We require a positive COVID-19 diagnosis because of PASC-like self-reported symptoms and physiological changes among COVID-19 untested/negative individuals.^26,41,46^ This is a decentralized trial, which allows for nationwide recruitment, increased participation of diverse and under-represented populations in research, and reduced participant burden.^140–142^

This study has several limitations. Sample size calculations are based on PASC surveillance data available when the study was launched. These data have since changed based on evolving definitions of PASC.^16^ Recruitment started after COVID-19 vaccines were rolled out irrespective of age or underlying medical conditions, which means our sample will include unvaccinated and vaccinated patients. The availability and effectiveness of COVID-19 vaccines and treatments as well as dominant variants throughout recruitment activities may affect study endpoints.^24,39,143,144^ Given limited evidence on demographic breakdown of patients who suffer from PASC, our sample reflects the US population by sex and race/ethnicity rather than PASC prevalence in population subgroups.^39^

The study application is available only in English. Thus, we exclude non-English speaking patients from participation. Self-selection bias is another limitation whereby participants with certain predispositions (e.g., digitally literate) may be inclined to enroll in the study. With a five-day enrollment window from a positive COVID-19 diagnosis, the initial severity of one’s illness can deter PRVs from enrolling. We have no baseline data from patients prior to their positive diagnosis. Given the decentralized nature of the study, its reliance on mobile technologies, and follow-up duration, certain limitations are expected (e.g., data loss due to technical issues or shipping delays, poor compliance, high attrition).^145–147^ The study wristband is not FDA approved. Physiological parameters do not capture all PASC symptoms (e.g., ageusia). Accrual is expected to fluctuate with infection rates, which may affect our ability to meet target sample or prolong recruitment.

## Figures and Tables

**Table 1:** Population by sex, race, and ethnicity in the US, 2019 estimates for 18- to 64-year-old adults and study sample (*N*= 550)

	US population (%)			Study sample (n)		
	Male	Female	Total	Male	Female	Total
Non-Hispanic or Latino						
White	50.09	49.90	73.04	164	163	327
Black	48.07	51.92	16.43	36	38	74
AIAN	48.77	51.22	1.69	3	4	7
Asian	47.78	52.21	8.46	18	20	38
NHPI	49.95	50.04	0.44	1	1	2
Total			100	222	226	448
Hispanic or Latino						
White	51.14	48.85	88.02	46	43	89
Black	48.88	51.11	5.72	3	3	6
AIAN	52.13	47.86	4.04	2	2	4
Asian	49.66	50.33	1.61	1	1	2
NHPI	51.62	48.37	0.58	1	-	1
Total			100	53	49	102

"Total” columns for US population represent %race of the total population by ethnicity (e.g., percent white of total non-Hispanic or Latino population). "Male” and "Female” cells for US population represent percent sex of the total population by race/ethnicity (e.g., percent males of total non-Hispanic or Latino Whites). Cells for the study sample show *n* for each racial/ethnic category by sex based on US population 18 to 64 years old for a sample of 550 patients.

**Table 2:** Schedule of activities

Procedures	Pre-enrollment	Enrollment; Day 0	Day 1-30	Day 31-60	Day 61-90	Day 91-120	Day 121-150	Day 151-180
Screening	x							
Informed consent		x						
Eligibility verification		x						
Baseline questionnaire		x						
Physiological data collection (# of days)			30x	8x	8x	8x	8x	8x
Ecological momentary assessments (# of EMAs)			30x	4x	4x	4x	4x	4x
Monthly survey (# of surveys)			1x	1x	1x	1x	1x	1x
Adverse outcomes survey (as applicable)			x-----------------------------x					
Technical/general support (patient- initiated)			x-----------------------------x					

**Table 3:** Sample study schedule

Month/data collection days	Frequency and timing of study activities		
	Device wearing	EMAs	Monthly survey

Montd 1:	Daily:	Daily:	Once:
Days 1–30	From 03–04-2023 to 04–02-2023	From 03–04-2023 to 04–02-2023	On 04–02-2023

Month 2:	4 times:	4 times:	Once:
Days 31, 32	From wake-up on 04–04-2023 to wake-up on 04–06-2023	On 04–06-2023	On 05–02-2023
Days 33, 34	From wake-up on 04–10-2023 to wake-up on 04–12-2023	On 04–12-2023	
Days 35, 36	From wake-up on 04–19-2023 to wake-up on 04–21-2023	On 04–21-2023	
Days 37, 38	From wake-up on 04–28-2023 to wake-up on 04–30-2023	On 04–30-2023	

Month 3:	4 times:	4 times:	Once:
Days 39, 40	From wake-up on 05–08-2023 to wake-up on 05–10-2023	On 05–10-2023	On 06–01-2023
Days 41,42	From wake-up on 05–16-2023 to wake-up on 05–18-2023	On 05–18-2023	
Days 43, 44	From wake-up on 05–20-2023 to wake-up on 05–22-2023	On 05–22-2023	
Days 45, 46	From wake-up on 05–28-2023 to wake-up on 05–30-2023	On 05–30-2023	

Month 4:	4 times:	4 times:	Once:
Days 47, 48	From wake-up on 06–04-2023 to wake-up on 06–06-2023	On 06–06-2023	On 07–01-2023
Days 49, 50	From wake-up on 06–09-2023 to wake-up on 06–11-2023	On 06–11-2023	
Days 51, 52	From wake-up on 06–14-2023 to wake-up on 06–16-2023	On 06–16-2023	
Days 53, 54	From wake-up on 06–28-2023 to wake-up on 06–30-2023	On 06–30-2023	

Month 5:	4 times:	4 times:	Once:
Days 55, 56	From wake-up on 07–06-2023 to wake-up on 07–08-2023	On 07–08-2023	On 07–31-2023
Days 57, 58	From wake-up on 07–10-2023 to wake-up on 07–12-2023	On 07–12-2023	
Days 59, 60	From wake-up on 07–20-2023 to wake-up on 07–22-2023	On 07–22-2023	
Days 61, 62	From wake-up on 07–25-2023 to wake-up on 07–27-2023	On 07–27-2023	

Month 6:	4 times:	4 times:	Once:
Days 63, 64	From wake-up on 08–04-2023 to wake-up on 08–06-2023	On 08–06-2023	On 08–18-2023
Days 65, 66	From wake-up on 08–12-2023 to wake-up on 08–14-2023	On 08–14-2023	
Days 67, 68	From wake-up on 08–19-2023 to wake-up on 08–21-2023	On 08–21-2023	
Days 69, 70	From wake-up on 08–24-2023 to wake-up on 08–26-2023	On 08–26-2023	

Schedule is based on an enrollment date of 03/01/2023. Participation in the study is for six months with a total of 70 days of digital device wearing.

In the first month, patients are required to wear a wristband and temperature patch, then only a wristband in months 2 to 6.

**Table 4:** Study measures

Domain	Eligibility verification	Baseline survey	Monthly surveys						EMAs	Adverse outcomes survey
			#1	#2	#3	#4	#5	#6		
Inclusion/exclusion questions	x									
Contact information	x									
Demographics	x	x								
Social determinants of health		x								
Technology access and acceptability										
Smartphone/tablet access	x	x								
Internet/ cellular data access	x	x								
Wearables acceptability			x					x		
COVID-19 information										
COVID-19 testing	x							x		
COVID-19 vaccination	x							x		
COVID-19 symptoms		x	x	x	x	x	x	x	x	
COVID-19 medications		x	x	x	x	x	x	x	x	
Medical information										
Concomitant medications		x								
Pregnancy and menstrual cycle		x								
Disability		x								
Existing and new health conditions		x	x	x	x	x	x	x		
Inpatient/outpatient care		x	x	x	x	x	x	x		x
Adverse events								x		x
Risk and protective factors										
Body measurements		x								
Fruit and vegetable intake		x								
Physical activity		x								
Tobacco use		x								
Alcohol consumption		x								
Marijuana use		x								
Prescription and illicit drugs use		x								
Patient-reported outcomes										
General health		x	x	x	x	x	x	x		
Perceived health change		x	x	x	x	x	x	x		
EQ-5D-5L health status *		x	x	x	x	x	x	x		
EQ-VAS										
PROMIS-29 Profile v2.1 health-related quality of life **		x	x	x	x	x	x	x		
PROMIS cognitive function v2.0 (short form 8a)		x	x	x	x	x	x	x		
PROMIS dyspnea severity v1.0 (short form 10a)		x	x	x	x	x	x	x		

* Captures 5 health dimensions: mobility, self-care, usually activities, pain/discomfort, anxiety/depression

** Captures 7 domains: physical function, anxiety, depression, fatigue, sleep disturbance, ability to participate in social roles and activities, pain interference, in addition to pain intensity

## Data Availability

Not applicable as no data were generated or analyzed for this study.

## Abbreviations

AIAN: American Indian and Alaska Native
BLE: Bluetooth Low Energy
BTRIS: Biomedical Translational Research Information System
COVID-19: Coronavirus disease 2019
EMA: Ecological momentary assessment
GBs: Gigabytes
HR: Heart rate
HRS: Hours
HRV: Heart rate variability
IC: Informed consent
EQ-5D-5L: EurQol 5 domains, 5 levels
EQ-VAS: EurQol visual analog scale
HZ: Hertz
LA: Los Angeles County
ME/CFS: Myalgic encephalomyelitis/chronic fatigue syndrome
NHPI: Native Hawaiian and Pacific Islander
NIDDK: National Institute of Diabetes and Digestive and Kidney Diseases
NIH: National Institutes of Health
NIMHD: National Institute on Minority Health and Health Disparities
OPR: Office of Patient Recruitment
PASC: Post-acute sequelae of COVID-19
PCR: Polymerase chain reaction
PHI: Protected health information
PII: Personal identifiable information
PPG: Photoplethysmography
PROs: Patient-reported outcomes
PROMIS: Patient-reported outcomes measurement information system
PRV: Prospective research volunteer
REDCap: Research Electronic Data Capture
RR: Inter-beat intervals between successive heartbeats
SARS-CoV-2: Severe acute respiratory syndrome coronavirus 2
SpO_2_: Peripheral capillary oxygen saturation
WHO: World Health Organization
